# A holistic approach to performance prediction in collegiate athletics: player, team, and conference perspectives

**DOI:** 10.1038/s41598-024-51658-8

**Published:** 2024-01-12

**Authors:** Christopher B. Taber, Srishti Sharma, Mehul S. Raval, Samah Senbel, Allison Keefe, Jui Shah, Emma Patterson, Julie Nolan, N. Sertac Artan, Tolga Kaya

**Affiliations:** 1 Department of Physical Therapy and Human Movement Science, Sacred Heart University, Fairfield, CT USA; 2 School of Engineering and Applied Science, Ahmedabad University, Ahmedabad, Gujarat India; 3 School of Computer Science and Engineering, Sacred Heart University, Fairfield, CT USA; 4 College of Engineering and Computing Sciences, New York Institute of Technology, New York, NY USA

**Keywords:** Predictive markers, Data processing

## Abstract

Predictive sports data analytics can be revolutionary for sports performance. Existing literature discusses players' or teams' performance, independently or in tandem. Using Machine Learning (ML), this paper aims to holistically evaluate player-, team-, and conference (season)-level performances in Division-1 Women's basketball. The players were monitored and tested through a full competitive year. The performance was quantified at the player level using the reactive strength index modified (RSImod), at the team level by the game score (GS) metric, and finally at the conference level through Player Efficiency Rating (PER). The data includes parameters from training, subjective stress, sleep, and recovery (WHOOP straps), in-game statistics (Polar monitors), and countermovement jumps. We used data balancing techniques and an Extreme Gradient Boosting (XGB) classifier to predict RSI and GS with greater than 90% accuracy and a 0.9 F1 score. The XGB regressor predicted PER with an MSE of 0.026 and an R^2^ of 0.680. Ensemble of Random Forest, XGB, and correlation finds feature importance at all levels. We used Partial Dependence Plots to understand the impact of each feature on the target variable. Quantifying and predicting performance at all levels will allow coaches to monitor athlete readiness and help improve training.

## Introduction

Coaching and training help athletes improve performance and win competitions. Skill-based training, strength and conditioning, and competition drive performance. To improve, the coaching team must understand and determine the type, amount, and training frequency^1,2^. Forecasting performance using Artificial Intelligence (AI)^3,4^ allows for optimized strategies and benefits stakeholders. These methods using large datasets can provide the coaches with robust feedback and help make informed decisions. Also, combining AI techniques with coaches’ expertise can improve prediction^5^.

Driven by big data and machine learning (ML), sports data analytics (SDA) has started to support evidence-based knowledge. In basketball, ML techniques are focused on players and the team, with performance prediction and injury risk as key challenges to be handled^6^. The ML approaches use supervised learning that builds models using input–output data pairs or unsupervised learning that identifies patterns using only input data^6^. The performance prediction is made using Artificial Neural Network (ANN), Decision Trees (DT) based Ensemble methods, and Support Vector Machine (SVM)^3,4^.

**Figure 1:**
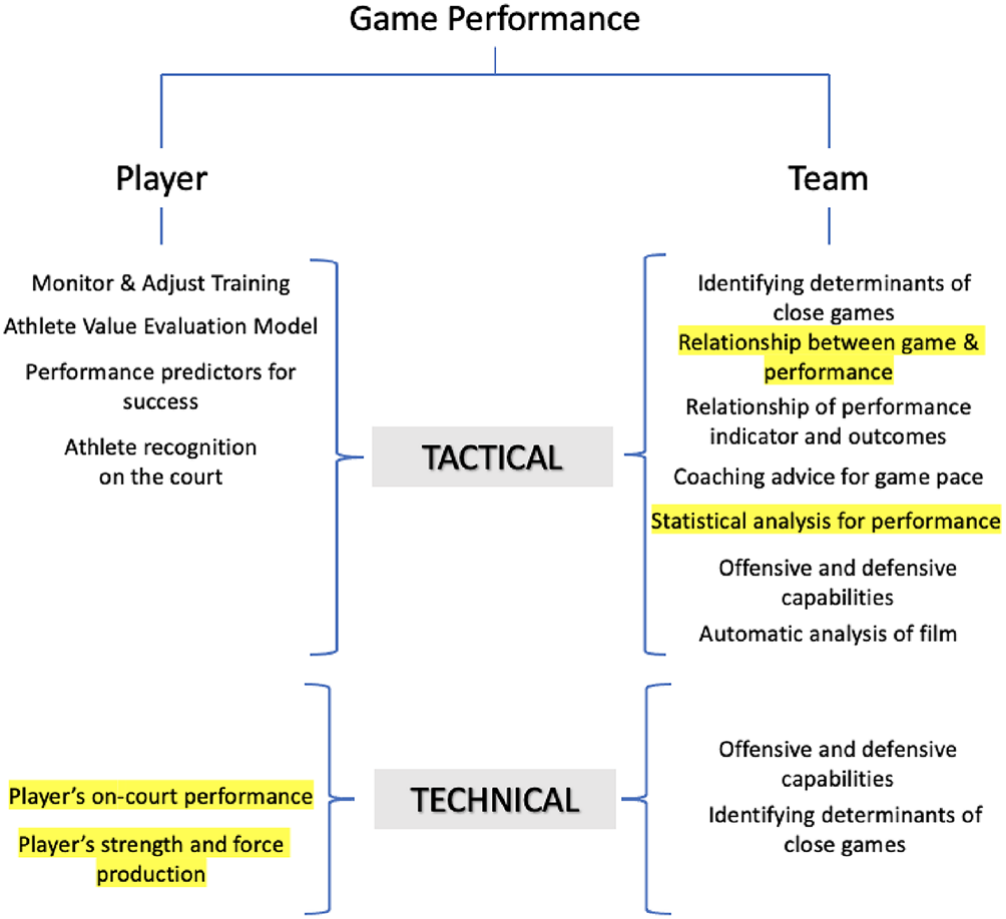
Graphical representation of how machine learning and statistical approaches help technical and tactical performance analysis of the player and team level. The factors highlighted in yellow are the methods used in this study.

Performance prediction is made using technical and tactical analysis, and factors shown in Fig. 1 are covered by ML techniques^3,6–11^ for performance prediction. The objective evaluation of the ML technique and coaches’ expertise significantly impacts player-level performance. Team-level performance can be used to evaluate individual games and team performance across the season or conference. A player’s importance is determined by measuring the average marginal contribution to winning a basketball game^10^. The method predicts winning probabilities associated with a selected lineup, and by averaging over many lineups, the player’s importance is estimated using Shapley values^10^. Players can also be ranked according to their contribution to the team’s performance using the Bayesian framework^12,13^. It is also necessary to build a predictive model that generalizes for individual athletes or the whole group^8^ with improved performance. Also, new features provide interesting insights into athletic performance by considering high sampling rate tracking systems^9^.

The coaches must know and adjust key factors, training variables, practice schedules, and overall stress to improve performance. Therefore, this paper uses a multi-level approach with supervised ML to analyze a competitive basketball season and predict individual, team, and whole-season performance.

## Methods

We define key performance indicators (KPIs) at each level to address the three-tiered question. At the tier 1—athlete level, we study their weekly readiness using countermovement jumps. The readiness was defined using KPI *reactive strength index modified* (RSImod), which is defined as follows (^14^):

1 $${\text{RSImod }} = JH/CT$$

where *JH* is the Jump Height and *CT* is the Contact Time. At the team level (tier 2), we calculate the *game score*^15^ for each game of the season. The game score KPI is computed using key game metrics and reflects the player’s in-game contribution. The KPI used at the conference level (tier 3) is *player efficiency rating* (PER); it shows a player’s efficiency in comparison to the average across the player’s conference^15^. The PER is computed after each game to account for scheduling difficulty during the season.

RSImod examined the effects of weekly training and stress on the athlete. We tied it with team-level KPI to examine if better readiness leads to better game scores. Finally, we tied level 2 and level 3 by checking if a greater game score across the season raised the PER.

**Figure 2:**
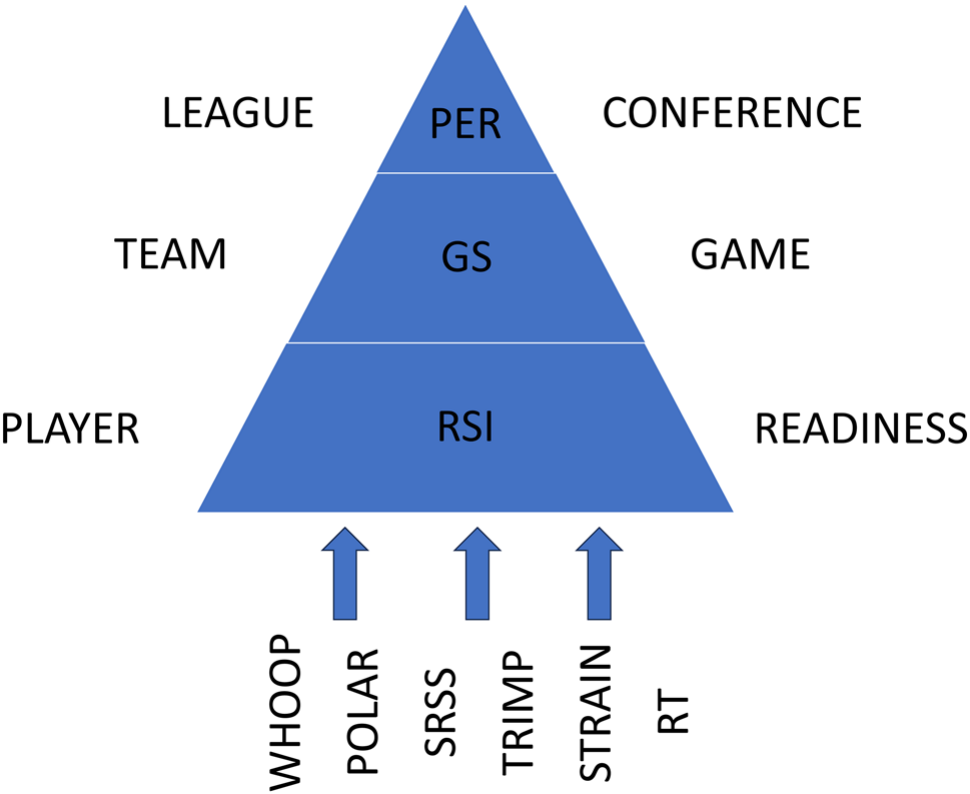
Experimental approach to the basketball performance prediction. At the player level, readiness is measured with all the data modalities in this study. The game score parameter measures team-level performance, whereas the Player Efficiency Rating evaluates conference-level performance. *SRSS* Short Recovery Short Stress, *TWLoad* Total Weekly Load, *RT* Resistance Training.

The three levels and each measured feature can be found in Fig. 2. Division-1 teams are categorized into conferences based on the region for collegiate athletics. Sacred Heart University’s Division-1 women’s basketball team is in the Northeast Conference (NEC) with eight other schools; therefore, PER is calculated based on NEC data. All conferences are under the National Collegiate Athletic Association (NCAA), considered the league.

### Participants and ethics

Sixteen Division -1 female basketball players (Age: 21 ± 3 yrs; Height: 174.21 ± 19.27 cm; Body Mass: 73.98 ± 11.52 kg) were tested and monitored between October 2021 and March 2022. A season’s analysis incorporated training and game workload measures, vertical jumps, subjective athlete questionnaires, sleep data, game score, and player efficiency rating parameters. This information was cleaned, organized, and analyzed with machine learning methods to find the critical features that predict performance. This project was submitted and approved by the University’s Institutional Review Board (IRB#170720A). The methods and procedures of the study were explained to the participants, and signed informed consent was obtained. All procedures followed the Declaration of Helsinki.

### Data collection

A sample of unidentified data used for the analysis (along with the Jupyter Notebook code file) is provided as additional Supplementary Data files.

#### Workload data

Training load was calculated as a weekly score by summing the total work completed in sports practice, metabolic conditioning, strength training, and gameplay. Following each training session, a session rating of perceived exertion (sRPE) was calculated based on a 1–10 Likert scale. The sRPE was completed by taking the total training time and the athletes' subjective rating. Total Weekly Load (TWLoad) and standard deviation were calculated using all sRPE values for the week. The weekly resistance training load was calculated by summing the total weight lifted during each session (sets x repetitions x load). Practice and game metrics (distance, heart rate, velocities, and accelerations) were calculated through the Polar Team Pro system (Polar Team Pro, Polar Electro, Kempele, FI) sampling at 10 Hz. All metrics were calculated using Polar’s proprietary collection and analysis software. Training monotony was calculated by taking the mean daily load and normalizing it by the weekly standard deviation of the training load. Training strain was calculated by taking TWLoad and multiplying it by the monotony score.

#### Vertical jump data

Countermovement Vertical jumps were collected once per week on the first practice day of each week (typically Monday or Tuesday). Subjects completed a standardized general warm-up in concert with practice. Then, they completed two vertical jumps of 50 and 75% of the athlete's perceived maximum with 30 s of passive rest between repetitions. Next, subjects would complete two maximal vertical jumps with a near-weightless polyvinyl chloride pipe placed below the C7 spinous process in the back-squat position to limit arm swing. The average of the two jumps was considered for analysis. All jumps occurred on dual force plates (FD Lites, Force decks, Newstead, QLS, AUS) sampling at 1000 Hz. All data were collected and analyzed in the proprietary Force Decks software. The metric of interest for this study was the RSImod, the KPI at the player level. Additional metrics collected were jump height via flight time and peak power, which were reported to the training staff as part of normal monitoring and testing of the athletes.

#### Subjective questionnaire data

Athletes were instructed to complete a bi-weekly recovery and stress questionnaire upon waking^16,17^. Each athlete individually completed eight questions (4 stress and 4 recovery questions). A 0–6 Likert scale was used for questions related to Negative Emotional State (NES), Overall Recovery (OR), Overall Stress (OS), Mental Performance Capability (MPC), Muscular Stress (MS), Physical Performance Capability (PPC), Emotional Balance (EB) and Lack of Activation (LA). This survey is valid and reliable for athletic populations^17^.

#### Sleep data collection

Whoop straps were distributed to all athletes and worn during the entire collection period. Athletes were instructed to wear them during sleep and daily activity, and data was collected through Whoop’s proprietary collection software. They were removed during games and practice. The metrics analyzed through the study were resting heart rate, heart rate variability, sleep parameters, and recovery parameters. In total, 22 features were collected and monitored daily for each athlete. The Whoop has been determined to be both reliable and valid compared to polysomnography in third-party testing for sleep and heart rate^18,19^.

#### Game score calculation

John Hollinger developed a metric, game score^15^, for calculating the athlete’s value per game. This being a compact measure of the productivity of the athlete as it quantifies the athlete's impact in a particular game, the game performance was quantified as *game score*. Please refer to Appendix I in the supplementary material.

#### Player efficiency rating calculation

PER is calculated using various factors, including points, rebounds, attempts, assists, steals, blocks, and turnovers^15,20^. Individual players’ names, the date, points scored, minutes played, 3-pointers attempted and made, 2-pointers attempted and made, free throws attempted and made, offensive rebounds, defensive rebounds, blocks, steals, turnovers, and personal fouls were exported from the university’s affiliated open-access athletic website into an Excel sheet^21^. The possession was then calculated for each team, allowing the League Pace to be found. The League pace was averaged by the game date and then averaged together to create the overall conference pace. Please refer to Appendix II in the supplementary material.

### Ethics approval

This project was submitted and approved by the University’s Institutional Review Board (IRB#170720A). The methods and procedures of the study were explained to the participants, and signed informed consent was obtained. All procedures were by the Declaration of Helsinki.

## Data analysis and prediction

There were missing data entries in the sleep, recovery, and questionnaire data. The missing data appeared to follow a Missing at Random (MAR) pattern, with a missingness rate of 13%. Therefore, we used the Multiple Imputation by Chained Equation (MICE) technique for imputing missing data, as it relies on conditional modeling of the missing feature with respect to available features^22^.

Multicollinearity analysis (correlation among parameters) revealed linear dependencies among features, causing bias in the ML model’s prediction. Simply dropping features would affect their impact on the game score. Using factor analysis (FA), we combined features having similar variance to obtain compact, lossless, and alternative representation. The features were modeled as a function of latent variables and combined in smaller groups known as factors^23,24^. ML techniques are then applied to factors that generate player, team, and conference-level predictions. Like any domain in which ML is applied, sports science also requires fairness, accountability, and transparency in decisions made by ML models. Feature importance explains ML decisions by assigning scores to factors impacting the game score^25^. It ranks them based on their influence on the game score and provides interpretability on the model’s predictions.

### Factor analysis

We observed linear dependency in some features, introducing redundancy and bias in the dataset. We performed factor analysis to discover latent factors for obtaining a lossless and compact feature representation. The in-game data from the Polar Band had 40 features on heart rate, distance, speed zones, recovery time, and accelerations, and FA resulted in 8 compact factors, as shown in Table 1.

**Table 1:** Categorization of polar data features using factor analysis that reduced the number of features from 40 to 8 (as factors).

Factors	Feature
*Speed and Total Acceleration Zones (F0)*	Number of accelerations: 2.99–2.00 (m/s^2^)
	Number of accelerations: 1.99–1.00 (m/s^2^)
	Number of accelerations: 0.99–0.50 (m/s^2^)
	Number of accelerations: 0.50–0.99 (m/s^2^)
	Number of accelerations: 1.00–1.99 (m/s^2^)
	Distance in Speed zone 1: 1.00–4.99 (km/h)
	Distance in Speed zone 2: 5.00–6.99 (km/h)
	Distance in Speed zone 3: 7.00–10.99 (km/h)
	Total distance (m)
*Average Speed and Distance (F1)*	Average speed (km/h), Distance (m/min), HR avg (bpm)
*Average Speed and Acceleration Zone (F2)*	Number of accelerations: 2.00–2.99 (m/s^2^)
	Distance in Speed zone 4: 11.00–14.99 (km/h)
	Distance in Speed zone 5: 15.00 (km/h)
	Number of accelerations: 50.00–3.00 (m/s^2^)
	Sprints
*Minimum Heart Rate (F3)*	HR min (bpm)
*Maximum Heart Rate (F4)*	HR max (bpm)
*Recovery Time (F5)*	Recovery time (h)
*Maximum Speed (F6)*	Maximum speed (km/h)
*High Intensity Acceleration Zone (F7)*	Number of accelerations: 3.00–50.00 (m/s^2^)

The factors are named based on the features they combined.

### Prediction

#### Player level

RSImod measures an athlete’s fatigue due to training and competition. Their readiness is measured using a countermovement jump test conducted at the beginning of the week. Athletes’ sleep and recovery patterns, training workload, and cognitive state in week N predict RSI for week N + 1. Using the quartile range of RSI, athletes were categorized into four groups or classes: Upper-performance group (U): RSI of 0.41 to 0.67, Upper-Middle performance group (UM): RSI of 0.36 to 0.41, Lower-Middle performance group (LM): RSI of 0.32 to 0.36, Lower performance group (L): RSI of 0.2 to 0.32.

**Figure 3:**
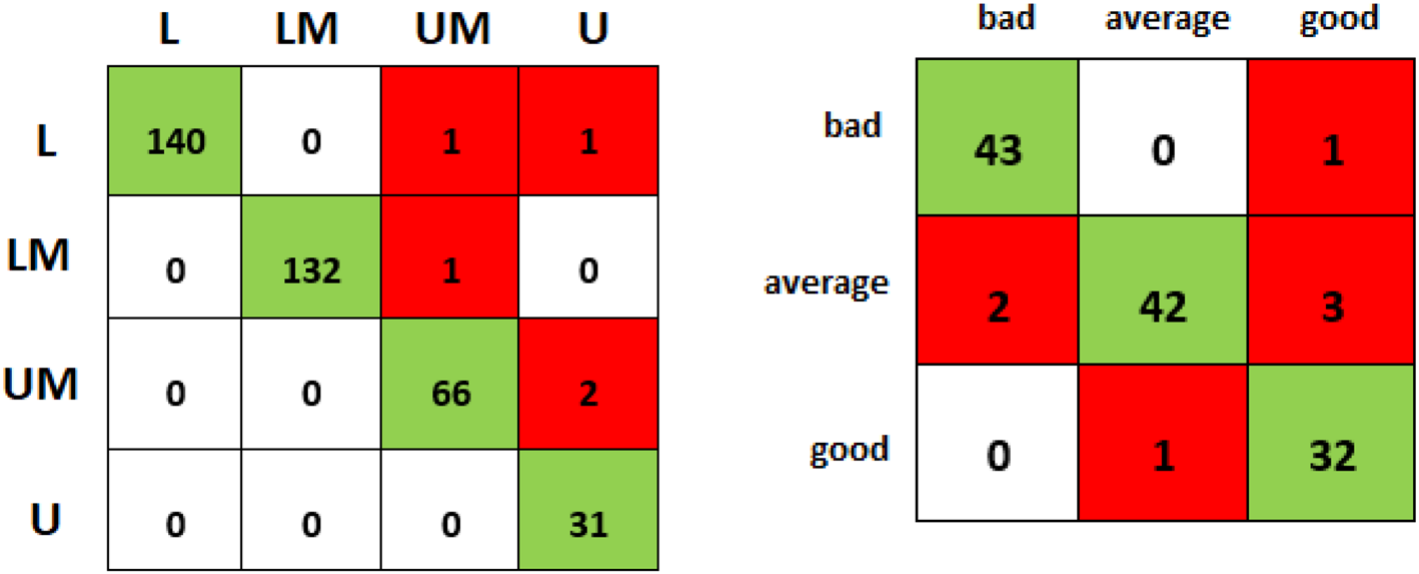
(**a**) (Left) Confusion matrix for the RSI prediction and the player-level performance KPI. Only 5 out of 291 predictions were incorrect (highlighted in Red). (**b**) (Right) Confusion matrix for the game score prediction; the KPI for the team-level performance. Green cells indicate correct predictions and red cells show misclassification. *U* Upper-performance group, *UM* Upper-Middle performance group, *LM* Lower-Middle performance group, *L* Lower performance group.

Fewer observations, 110 in U + UM compared to 181 in the LM + L group, resulted in data imbalance. Therefore, the synthetic minority oversampling technique (SMOTE) is used for oversampling the minority (U + UM) classes and balancing the dataset^26^. The eXtreme Gradient Boosting (XGB) classifier was used as it is efficient, flexible, portable, robust to outliers, and has superior regularization capabilities^27^. The training and test sets are divided into 70 (1106 records): 30 (374 records) splits. The training set fits the model, while the test set evaluates the model's performance. The XGB classifier, while predicting RSImod, had an accuracy of 98.67% and an F1 score of 0.986. Figure 3a represents the confusion matrix for predictions. Diagonal (highlighted in green) refers to the correct predictions, and off-diagonal shows an incorrect prediction. Please refer to Appendix III in the supplementary material for formulas. We have assessed the uncertainty of XGB by fixing the seed to ensure reproducibility and consistency, using the K fold cross-validation technique^28^, which observes prediction variability across data splits and measures standard deviation in feature importance scores. Detailed analysis of the uncertainty experimentation is provided in Appendix IV.

#### Team level

Athlete’s game performance was quantified at the team level by the game score^15^. It is an in-game statistic and reflects an athlete's contribution to the team. We have predicted game scores using previous weeks’ sleep, training, questionnaire^17^, jump, and in-game statistics (measured using the polar unit). Using k-means clustering over a game score dataset, it is divided into three clusters—bad, average, and good. We observed that generating synthetic samples from the minority class does not always work well as it does not account for the complete data variability. Therefore, we used a combination of over (SMOTE) and undersampling (ENN) techniques in the season for data balancing. Appendix V discusses the various under and over-sampling techniques and their performance. Using train-to-test splits of 70 (87 records): 30 (37 records), and stratified K-fold cross-validation, the XGB classifier for predicting the class of the game score provided an accuracy of 94.20% and an F1 score of 0.94. Figure 3b depicts the confusion matrix for game score prediction.

#### Conference-level

Player efficiency rating evaluates an individual's efficiency compared to the average across the conference^15^. It was predicted using sleep and recovery input features, training, subjective stress, reactive strength, and in-game statistics. We used the XGB regressor to predict PER as a continuous variable. The 70:30 train test splits resulted in 84 records for training and 35 for testing. With K fold cross-validation, the XGB regressor provided a Mean Squared Error (MSE) of 0.026 (ideal ⁓ 0) and R^2^ of 0.68 (ideal ⁓ 1). Please refer to Appendix III in the supplementary material.

### Feature importance

We devise an ensemble-based feature importance approach using scores from Random Forest (RF), XGB classifier/regressor, and correlation (CORR) to yield robust results^27^. In RF and XGB, feature importance Gini index (GI), which measures node impurity, is used to compute feature importance. The important feature causes a decrease in the value of GI and allows their selection. The RF and XGB determine the feature's contribution to prediction and their feature importance. The CORR also determines feature importance by finding their contribution to target prediction, but it generates a value between − 1 and 1. We utilized a nonlinear weighted average technique to aggregate the XGB, RF, and CORR feature importance scores. This allowed us to customize weighing, leverage the advantages of different methods, and provide a balanced assessment of feature importance (see Appendix VI).

#### Player

Appendix VII (Fig. S2) in supplementary material represents the importance of the explanatory features for the target feature Reactive Strength Index, a player-level KPI. It was predicted using three modalities—sleep and recovery, training, in-game, and subjective stress. The top five features contributing to RSI prediction are Training Strain, RT Volume Load, TWLoad—all three from the training modality, HRV from the sleep and recovery modality, and MPC subjective stress modality. It is important to note that the top three features of RSI are from the training data.

#### Team

Appendix VII (Fig. S4) in supplementary represents the feature importance for the target feature Game Score, a team-level KPI. The input data for game score prediction comes from modalities—reactive strength, in-game statistics, sleep and recovery, subjective stress, and training^29^. The top five features for game score prediction are Average Speed and Distance (Factor F1), Recovery Time—in-game statistics modality, Daily Average—training modality, Speed and Total Acceleration Zones (F0), and High-Intensity Acceleration Zone (F7) again from in-game statistics modality. One can observe that the most important features of game scores are derived from in-game statistics.

#### Conference

Player efficiency rating is a conference-level KPI used in the present work. Appendix VII (Fig. S5) in the supplementary document represents its feature importance. Again, they are derived from five modalities—reactive strength, in-game statistics, sleep and recovery, subjective stress, and training. The top five features significantly contribute to PER prediction: Peak Power—reactive strength modality, Maximum Speed—in-game statistics, Sleep consistency, Deep Sleep Hours—sleep and recovery modality, and Emotional Balance (EB)—subjective stress modality. It can be observed that unlike player level and team level, no category dominates in feature importance.

## Discussion

This study predicts performance at three tiers—player, team, and conference using ML. The KPIs at each level and their interactions across levels provide coaches insight into how to prepare for training and manage workloads during the competitive season. We use partial dependence plots (PDPs) to interpret the impact of a feature on the performance^30^. PDP captures the instantaneous change in a feature over the target while holding all other features constant.

### Athlete level (RSImod prediction)

RSImod positively correlated with training metrics, physiological measurement by WHOOP, and subjective stress. Adequate stimulus across modalities allows athletes to show better readiness for the following week. The appropriate amount of strain with proper training strategies increased the RSImod, as shown by the PDP of the strain in Appendix VIII (Fig. S5). However, when strain increases beyond the point of positive adaptation, it can result in fatigue, impaired recovery, and reduced readiness associated with overtraining. Therefore, when overtraining is present, it can lead to stagnation in the RSImod. We found a decreased RSI due to an increased RT volume load due to overtraining. The increased RT volume load also results in a plateauing effect due to a longer recovery time. The PDP for TWLoad signifies that we must avoid too little or too much training as it negatively impacts RSImod. Evidence from sports science suggests the negative impact of excessive training load on RSImod^31^. The heart rate variability indicates physiological readiness, and its greater value signifies better adaptability^14^. Providing feedback on HRV improves athlete performance^32^, and the intensity and RT volume load negatively impacts HRV^33,34^. MPC’s fifth most important feature is the subjective stress category, which provides insights into how an athlete copes with demands. Thus, all five factors contribute to the athlete’s readiness for the coming week.

We performed a time series analysis and predicted the N + 1 week’s RSI score using the past N weeks' data. The best accuracy (70%) and F1 (0.71) were observed for week 17. However, we could not observe consistency as some players contracted COVID-19 and missed the practice season. For the upcoming season, we will improve methodology with robust data collection and refine time series analysis.

### Team level (game score prediction)

The motor abilities of athletes during the game, like jumping, sprinting, accelerating, changing directions, and decelerating, reflect their strength, endurance, and speed^29^. From a technical and tactical viewpoint, these factors impact the athlete’s game performance. Four of the five most important features (average speed and distance, recovery time, speed and total acceleration zones, and high-intensity acceleration zones) are in-game motor abilities. Usually, high-scoring athletes cover lesser distances but achieve top speeds during the games^29^. The most important factor—Average speed and distance (F1) includes the maximum speed achieved by an athlete during a game, as shown in the PDP of Appendix VIII (Fig. S6). An athlete in high speed indicates high-intensity moments like shooting or scoring^31^, which suggests the athlete is recording more time in high-intensity acceleration zones^35^. It increases the probability of an athlete achieving a greater game score. Acceleration profiles may vary among players and throughout a game^36^. Higher accelerations were found in the game and during key moments when the match was tightly contested^36,37^.

Recovery time depicts physical preparedness and is related to overall fitness. More time spent in recovery prevents acute spikes in workload, resulting in less fatigue, improved game performance (as shown in PDP), and injury prevention^38^. The daily average is representative of the total workload, encompassing physical training, metabolic stress, sports practice, and competition. When extrapolating from the player level, the daily load is related to the amount of training. Consistent exposure to the correct amount of training provides better individual readiness and impacts game scores (as shown in PDP). This implies that coaches and training staff should examine the global workload incurred by each athlete during preparation and competition.

### Conference level (player efficiency rating prediction)

Interestingly, peak power emerged as the main predictor of the PER (Appendix VIII, Fig. S7). However, previous research has demonstrated that power output is a discriminator of players within a team and between competitive levels^39^. In this context, peak power was derived from a countermovement jump used for weekly monitoring. This may provide insights into which athletes are better prepared each week and the need for fatigue management. Next, maximum speed depicts high-intensity moments in competitions. Maximal intensity moments in a match (shooting or scoring) lead athletes to higher speed zones. Hence, the maximum speed of athletes recorded during competitions positively correlates with their conference performance^40^. The following two predictors come from the Whoop strap data set. Deep sleep hours are when athletes recover from the stress they undergo during training and competition. Sleep consistency is the rating of how regular the sleep patterns of the athlete are over the days of the week. Previous studies have shown that sleep extension improved basketball performance^41,42^. Greater sleep hours and more time spent in deep sleep may provide recovery as this is the time the body repairs itself and provides the most restorative sleep^43,44^. Finally, emotional balance is a subjective feature reflecting the recovery/stress of the athlete from the overlapping demands of training, competitions, and academic pressure imposed upon them. Across the season, emotional balance is likely to reflect the recovery and stress an athlete is experiencing and be viewed as a cumulative aspect of the athletic preparation process.

### Most important modality at each level

**Figure 4:**
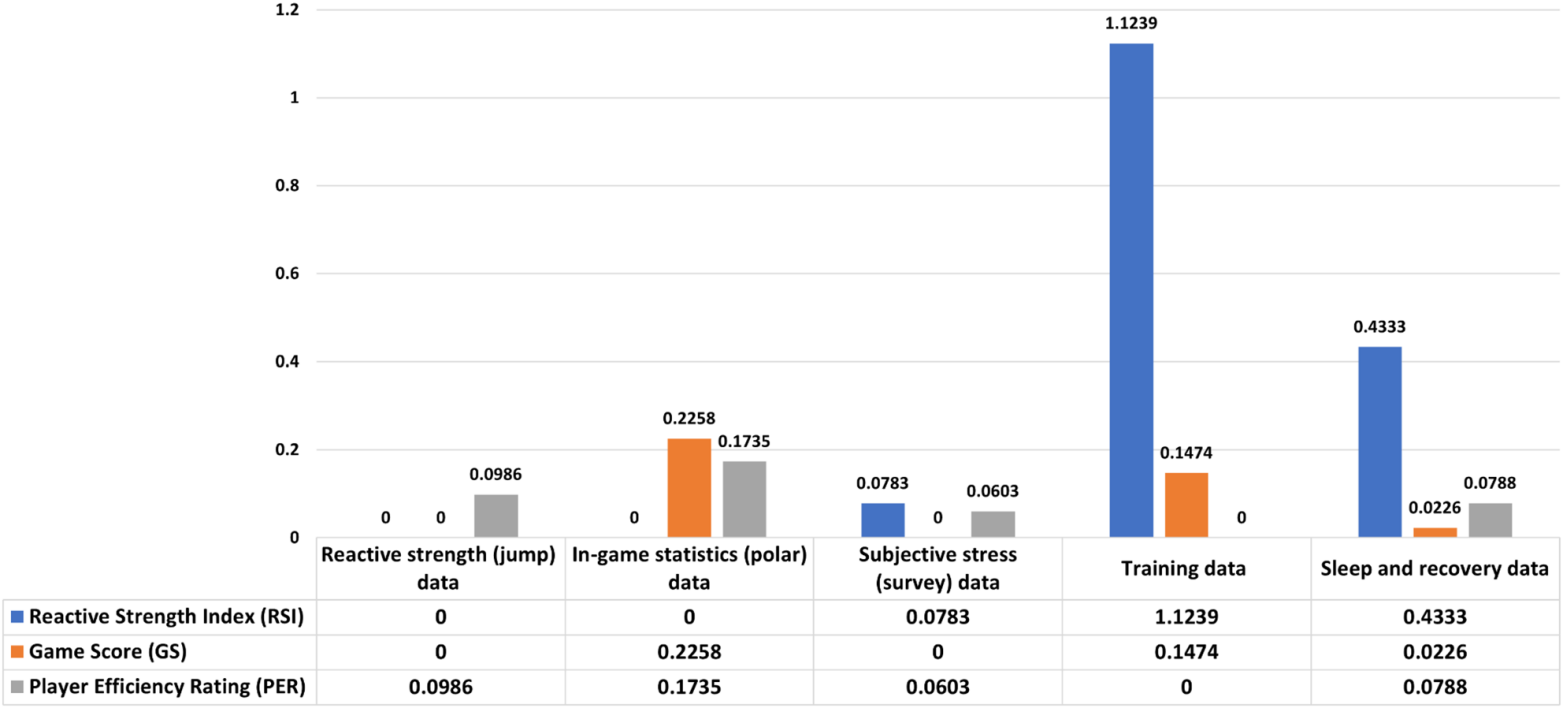
Most important modality analysis on which parameter groups contributed to the prediction the most in each category.

This paper uses data from five modalities—reactive strength, in-game statistics, sleep and recovery, subjective stress, and training to predict metrics at three levels. We added each modality's ten most important features to find the most important modality (MIM) for RSImod, Game score, and PER. It can be observed from Fig. 4 that training, sleep, recovery, and subjective stress impact RSImod and MIM's training data. The game score is impacted by in-game statistics, training, and sleep and recovery data, and MIM is in-game statistics. PER is impacted by in-game statistics, sleep and recovery, subjective stress, and reactive strength, with MIM as in-game statistics.

### Strengths and limitations

Detailed analysis is possible as data for the whole team is available during preparation and competitive periods. These differences in training can be observed for off, pre, and competitive seasons. The quantification of internal and external metrics allowed for analyzing athletes’ responses to training and competitive stresses. ML techniques revealed the most important modalities that help optimally prepare athletes for competition. The combination of three perspectives—player, team, and conference spanned from micro to medium to macro exploration. The most important features are selected at each level for analysis and prediction. Sports scientists can use the feature importance to make an informed decision.

The paper studies a homogeneous group of female basketball players, and the model may not generalize across sports or genders. This project only encompassed one year of data, and more is needed for the model to work across seasons with changing rosters and changes in the competitive schedule. The analysis on each level was based on a single metric (RSI, Game Score, or PER), which may limit understanding of the impact on performance. Although the data imbalance was handled, data biases due to using a particular data imputation technique or bias in measurements due to gender would need to be addressed.

### Recommendations for practice and research

Coaches and practitioners should attempt to collect multiple data streams to make informed decisions about training, sports practice, and competition. By identifying KPIs at various levels of performance, practitioners can monitor athletes in the long term and make key changes when necessary to help the athletes better prepare for competing (and sometimes conflicting) demands. Real-time dashboard applications can provide timely feedback to coaches to make informed decisions (see Appendix IX for an example)^45^. The current research focused on identifying the key performance indicators to hypothesize how they can help to improve performance. Our future work will test these hypotheses and see how well they help improve athletic performance. No metrics should be considered in isolation, but part of a well-rounded monitoring program that provides actionable information for sports coaches, strength coaches, and sports scientists. Future research should attempt to quantify key metrics in other sports of various levels to create prediction and modeling that fits that sport's requirements.

There are emerging metrics that monitor cumulative workload’s influence over time for better imputation and analysis^46^. Adjusting the decreasing parameter allows the coach consistent monitoring even with many observations missing in the dataset^46^, which could be implemented for future work. The data and algorithm debiasing techniques may be used to improve the fairness of the approach. The XAI approach may have coach and player centricity with explanations in the sports science vocabulary. Time-series approaches can also be incorporated to predict performance for the following week using the previous weeks’ data, which will also be the future work for this research group (current progress on the time-series explorations is summarized in Appendix X). Similarly, game performance for the following game could be predicted using the previous games’ data.

## Conclusion

Athletes' performance was predicted at the individual, team, and conference levels for a fine-grained assessment of the impact of different modalities at different levels. Quantification and explanation of these predictions would provide actionable insights to coaches for continuously monitoring athlete readiness and guided training (based on individual level performance), deciding team composition for the coming matches (based on team level performance), and identifying the most valuable player of the conference—strategizing for the coming season (based on conference level performance).

### Supplementary Information

Supplementary Information 1.

Supplementary Information 2.

Supplementary Information 3.

## Data Availability

Data cannot be made available publicly because participants can be identified by cross-referencing the university’s athletic website (the game score parameter was obtained from publicly available data). However, a sub-portion of the data that is de-identified was made available. Contact the corresponding author, Dr. Tolga Kaya, for further inquiries about the data.

## Code availability

Jupyter Notebook was included as a Supplementary file.
