# Supplementary material for: A holistic approach to performance prediction in collegiate athletics: player, team, and conference perspectives
Source: Sci Rep. 2024 Jan 12;14:1162. doi: 10.1038/s41598-024-51658-8 (PMC10786827; doi:10.1038/s41598-024-51658-8)
Supplement: Supplementary file 1 — Supplementary Information 1. [file 41598_2024_51658_MOESM1_ESM.docx]

**Supplementary Material**

**A Holistic Approach to Performance Prediction in Collegiate Athletics: Player, Team, and Conference Perspectives**

Christopher B. Taber^1^*, Srishti Sharma^2^*, Mehul S. Raval^2^, Samah Senbel^3^, Allison Keefe^1^, Jui Shah^1^, Emma Patterson^1^, Julie Nolan^1^, N. Sertac Artan^4^, Tolga Kaya^3^**

* Equal contribution

** Corresponding author

Tolga Kaya

[kayat@sacredheart.edu](mailto:kayat@mail.sacredheart.edu)

1. Department of Physical Therapy and Human Movement Science, Sacred Heart University, Connecticut, USA
2. School of Engineering and Applied Science, Ahmedabad University, Gujarat, India
3. School of Computer Science and Engineering, Sacred Heart University, Connecticut, USA
4. College of Engineering and Computing Sciences, New York Institute of Technology, New York, USA

# Appendix I - Game Score Calculation

Hollinger Game Score was derived from athlete’s game statistics such as assists (AST), blocks (BLK), field goals (FG), field goal attempts (FGA), free throws (FT), free throw attempts (FTA), offensive (ORB) and defensive rebounds (DRB), personal fouls (PF), points scored per game (PTS), steals (STL) and turnovers (TOV) [(14)](https://www.zotero.org/google-docs/?a9m1o3).

*
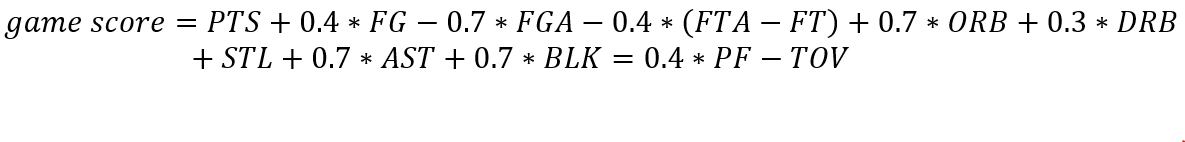
*

# Appendix II - PER Computation

Player Efficiency Rating (PER) is used in basketball to show an individual player’s efficiency compared to the average across the player’s league (1). It is calculated using a series of different factors, including points, rebounds, attempts, assists, steals, blocks, and turnovers (1). The equation considers whether the action is positively contributing to the game, such as scoring, or negatively affecting the game, such as turning over the ball (1). The equation also factors in the pace of the league, which shows the amount of possession the team has during a game compared to the total minutes played (1). When calculating player efficiency rating, the first type of PER calculated is known as the unadjusted PER (uPER), meaning that it has not been adjusted in comparison to the other players within the conference (Hamalian). The adjustments are necessary to have a fair scale to compare the players across the conference (2). The uPER is then adjusted using the league pace to create a comparison for the conference for that season (2). Finally, it is adjusted using the average PER of a player to create a comparison that can be used for any two players in the sport (2).

Individual players’ data were exported from the Sacred Heart University website (3) into an Excel sheet to calculate PER. The individual player’s name, the date, points scored, minutes played, 3-pointers attempted and made, 2-pointers attempted and made, free throws attempted and made, offensive rebounds, defensive rebounds, blocks, steals, turnovers, and personal fouls were recorded. This was repeated for all 16 players.

An Excel spreadsheet was then made to show the same statistics for the league with ten other teams. The data was abstracted from each team’s website. For every week, the totals and averages were calculated for the ten teams in the following categories: points, minutes, field goals made and attempted, points made and attempted, free throws made and attempted, rebounds, assists, blocks, steals, turnovers, points for, offensive rebounds, and defensive rebounds. For each game, the total minutes were inputted at 200 unless the game went into overtime. Dates where teams did not play were marked by making the cells black. Possession (POSS) was then found for each team using the following equation:

POSS = 0.5*((FGA + 0.4*FTA-1.07*(ORB/(ORB+Opp_DRB))*(FGA-FGM+TO)+(Opp_FGA+0.4*Opp_FTA-1.07*(Opp_ORB/(Opp_ORB+DRB)) * (Opp_FGA - FGM) + Opp_TO)))

FGA: Field Goals Attempts

FTA: Free Throw Attempts

ORB: Offensive Rebound

Opp_DRB: Opponent Defensive Rebounds

FGM: Field Goals Made

TO: Turnovers

Opp_FGA: Opponent Field Goal Attempts

Opp_FTA: Opponent Free Throw Attempts

Opp_ORB: Opponent Offensive Rebounds

DRB: Defensive Rebounds

Opp_TO: Opponent Turnovers

The League Pace (lg_Pace) was then found by applying the following formula to each individual team:

lg_Pace =40*((POSS+Opp_Poss)/(2*Min/5))

This was then averaged by game date. The League pace for each week was then averaged together to create the overall league pace. Then, using R, we calculated the unadjusted PER (uPER) for each player for every game. This was calculated using the following formula:

uPER = (1 / MP) *

[ 3P

+ (2/3) * AST

+ (2 - factor * (team_AST / team_FG)) * FG

+ (FT *0.5 * (1 + (1 - (team_AST / team_FG)) + (2/3) * (team_AST / team_FG)))

- VOP * TOV

- VOP * DRB% * (FGA - FG)

- VOP * 0.44 * (0.44 + (0.56 * DRB%)) * (FTA - FT)

+ VOP * (1 - DRB%) * (TRB - ORB)

+ VOP * DRB% * ORB

+ VOP * STL

+ VOP * DRB% * BLK

- PF * ((lg_FT / lg_PF) - 0.44 * (lg_FTA / lg_PF) * VOP) ]

Whereas,

factor = (2 / 3) - (0.5 * (lg_AST / lg_FG)) / (2 * (lg_FG / lg_FT))

VOP = lg_PTS / (lg_FGA - lg_ORB + lg_TOV + 0.44 * lg_FTA)

DRB% = (lg_TRB - lg_ORB) / lg_TRB

Once calculated, the resulting number was entered into an Excel sheet which indicated the player, the date, and the uPER. The uPER was then converted to the average PER (aPER), by multiplying the uPER by the Pace Adjustment. The pace adjustment was calculated using the following formula:

aPER = lg_Pace/team_Pace

From there, the adjusted League PER (lg_aPER) was calculated using the following formula:

aPER*(33/Lg_aMIN)

Lg_aMin indicated the average minutes played across all players in the league, which was calculated to be 21.0919.

The average lg_aPER was then found. From there, we calculated the PER for each player using the following equation:

PER = aPER*(10/lg_aPER)

The factor of 10 was used because the average used for professional men’s basketball resulted in numbers outside the reference frame. The factor is the average PER for the league, so in collegiate women’s basketball, it should be lower than in a professional men’s setting.

Using 10 as the factor resulted in an average PER of 7.8 for all 16 players throughout the season. Using the NBA PER Reference Guide (4), an average of 7.8 would have the team in the “Player who won’t stick in the league” ranking.

This would make sense, as there is high variability within the PER. Players who only had a few minutes and had only negative results had very low PERs, with the lowest being calculated at -147.9. This player was in the game for one minute, and during that time, they had two turnovers and one personal foul, making their contributions entirely negative in a short period.

The highest PER was calculated at 53.5, with the player having played 31 minutes and scoring 24 points. While they did have negative contributions, the increased playing time, as well as the amount scored, resulted in the high PER.

From the season's first game to the last, the team average PER decreased from 17.5 to 14.1. This change may be due to the final game being a tournament game, with higher stakes for both teams involved. When looking at the last regular season game the score still decreases, but only from 17.5 to 17.2, which is much less of a significant difference.

The average team PER for each game is higher for wins than it is for losses; however, it is important to note that the highest team average PER of 20.6 was from a game that resulted in a loss. The lowest team average PER of -11.3 also resulted in a loss. Given more than two times as many losses as there were wins, the average PER for the season of 7.8 more closely reflected the average loss PER of 6.6 than in comparison to the average win PER of 12.0.

# Appendix: III Prediction Formulas

# Appendix IV - Uncertainty

We have assessed uncertainty by fixing the seed of Random Forest and XGB and using the K fold cross-validation technique.

We set the seed using the NumPy library while implementing Random Forest-based and XGBoost-based feature importance analysis to ensure consistency and reproducibility. These approaches introduce randomness through bootstrapping and feature selection, leading to varying feature importance scores across runs. By fixing the seed, we made the randomness consistent across runs, giving reproducibility, consistency, model comparisons and sharing, and debugging. It also allowed for finding the source of variability and attributing differences in feature importance or model performance to actual changes rather than random chance.

We used 10-fold cross-validation to evaluate feature importance in both RF and XGB. After splitting data, training models, and calculating the feature importance within each fold, the aggregate feature importance score was calculated to identify the most influential features, ensuring robust and reliable feature ranking. We compute the standard deviation to quantify uncertainty, and the following three Tables S1 to S3 show uncertainty for the top five features at all three levels. From these tables, the top 5 features for each level of performance, as predicted by both techniques, are the same, with minor differences in the ordering and variance. For example, TWLoad is the third most significant feature per XGB, while MPC per RF. The importance score, however, for TWLoad is 0.037+0.00002 (XGB) and 0.035+0.00077 (RF), and for MPC, it is 0.034+0.00037 (XGB) and 0.036+0.00181 (RF), which shows consistency across the 10-folds as well as the two feature importance scores.

(Attaching Tables S1, S2, and S3 with Feature Importance (FI)+Standard Deviation (SD in appendix)

Table S1: Uncertainty in FI of Top - 5 RSI features.

| RSI Top 5 Features | XGBoost based Feature Importance (FI+SD) | Random Forest based Feature Importance (FI+SD) |
| --- | --- | --- |
| Training strain | 0.051+0.00007 | 0.048+0.00231 |
| RT Volume Load | 0.048+0.00061 | 0.052+0.00010 |
| TWLoad | 0.037+0.00002 | 0.035+0.00077 |
| HRV | 0.036+0.00018 | 0.036+0.00060 |
| MPC | 0.034+0.00037 | 0.036+0.00181 |

Table S2: Uncertainty in FI of Top - 5 Game Score features.

| Game Score Top 5 Features | XGBoost based Feature Importance (FI+SD) | Random Forest based Feature Importance (FI+SD) |
| --- | --- | --- |
| Average speed and distance (F1) | 0.070+0.00030 | 0.064+0.00441 |
| Recovery time | 0.061+0.00121 | 0.069+0.00012 |
| Daily average | 0.058+0.00197 | 0.056+0.00001 |
| Speed and total acceleration zones (F0) | 0.040+0.00113 | 0.042+0.00027 |
| High intensity acceleration zones (F7) | 0.037+0.00020 | 0.034+0.00320 |

Table S3: Uncertainty in FI of Top - 5 PER features.

| PER Top 5 Features | XGBoost based Feature Importance (FI+SD) | Random Forest based Feature Importance (FI+SD) |
| --- | --- | --- |
| Peak power | 0.058+0.00172 | 0.067+0.00135 |
| Maximum speed | 0.054+0.00110 | 0.068+0.00020 |
| Sleep consistency | 0.044+0.00386 | 0.050+0.00518 |
| Deep sleep hours | 0.038+0.00174 | 0.049+0.00173 |
| Emotional balance | 0.033+0.00007 | 0.042+0.00684 |

# Appendix V Imbalance

We observed that generating synthetic samples from the minority class does not always work well as it does not account for the complete data variability. We found that a combination of over and undersampling works best in the proposed approach.

While SMOTE remains a dependable method for balancing data, it's worth noting that the synthetic samples it generates exhibit linear correlations with their parent samples. Consequently, a combination of SMOTE with an undersampling technique yields superior results compared to either standalone oversampling or undersampling methods, as mentioned in (5). We tried several combinations of undersampling techniques with SMOTE.

ADASYN, a variant of SMOTE, introduces adaptiveness into the classification boundary to address challenging instances. However, it can occasionally overestimate the difficulty of minority samples and exhibits sensitivity to the data distribution, as discussed in (5). Borderline SMOTE contributes to establishing the inter-class boundary, but its effectiveness is closely tied to the dataset's specific characteristics and the distribution of minority class instances, as mentioned in (5). SMOTE combined with Tomek links and SMOTE with Edited Nearest Neighbor (ENN) techniques are recommended for a more comprehensive approach. ENN and Tomek Links are undersampling techniques and they effectively reduce sample overlap between different classes, rectify data imbalance issues, and mitigate overfitting concerns, as highlighted in (6).

We finalized SMOTE in combination with the ENN (Edited nearest neighbors) technique. SMOTE synthesizes new instances from the minority class (oversampling). It may introduce noise as it increases the probability of class overlap. Hence, we apply the ENN data balancing technique over the oversampled dataset as a selective method that removes examples from the majority class whose neighbors, identified by the k-nearest neighbor (k-NN) algorithm, belong to a different class (6). A summary of these combined methods is listed in Table S4.

Table S4: Impact of different data balancing techniques. SMOTE (Oversampling) and ENN (Undersampling) is the best choice for the proposed work.

| Data Balancing Technique | Player Level  (RSI level prediction) | | Team Level  (Game score prediction) | | Conference Level  (PER prediction) | |
| --- | --- | --- | --- | --- | --- | --- |
|  | Accuracy | F1 score | Accuracy | F1 score | MSE | R2 |
| SMOTE | 73.48% | 0.74 | 76.24% | 0.76 | 0.103 | 0.07 |
| SMOTE+ADASYN | 82.63% | 0.82 | 78.76% | 0.77 | 0.870 | 0.26 |
| SMOTE+Borderline SMOTE | 88.27% | 0.89 | 84.52% | 0.85 | 0.230 | 0.45 |
| SMOTE+Tomek link | 82.25% | 0.82 | 77.67% | 0.77 | 0.380 | 0.33 |
| ***SMOTE+ENN*** | ***98.67%*** | ***0.98*** | ***94.20%*** | ***0.94*** | ***0.026*** | ***0.68*** |

# Appendix VI - Importance Aggregation

We used a weighted average technique to aggregate the XGB, RF, and CORR feature importance scores. It allows us to customize weighing, leverage the advantages of different methods, and provide a balanced assessment of feature importance.

Fig. S1 illustrates the feature aggregation followed in the paper. While XGB and RF assign a score from 0 to 1, CORR gives a score from -1 to 1. We scaled the CORR scores from range -1 to 1 to range 0 to 1. Then, for each of these techniques, we selected the top 15 features and fit a random forest model to predict the target feature. For RSI and GS, we used the model F1 score, and for PER, we used the model MSE as the performance metric for comparing the efficacy of the three feature importance techniques. The performance metric values [CORR, XGB, RF] were fed to a Softmax function, generating a probabilistic score we used as weights for the respective techniques. An aggregate feature importance score was calculated, taking the weighted average of the feature importance score generated by the three methods. We then ranked features based on the aggregate importance score.


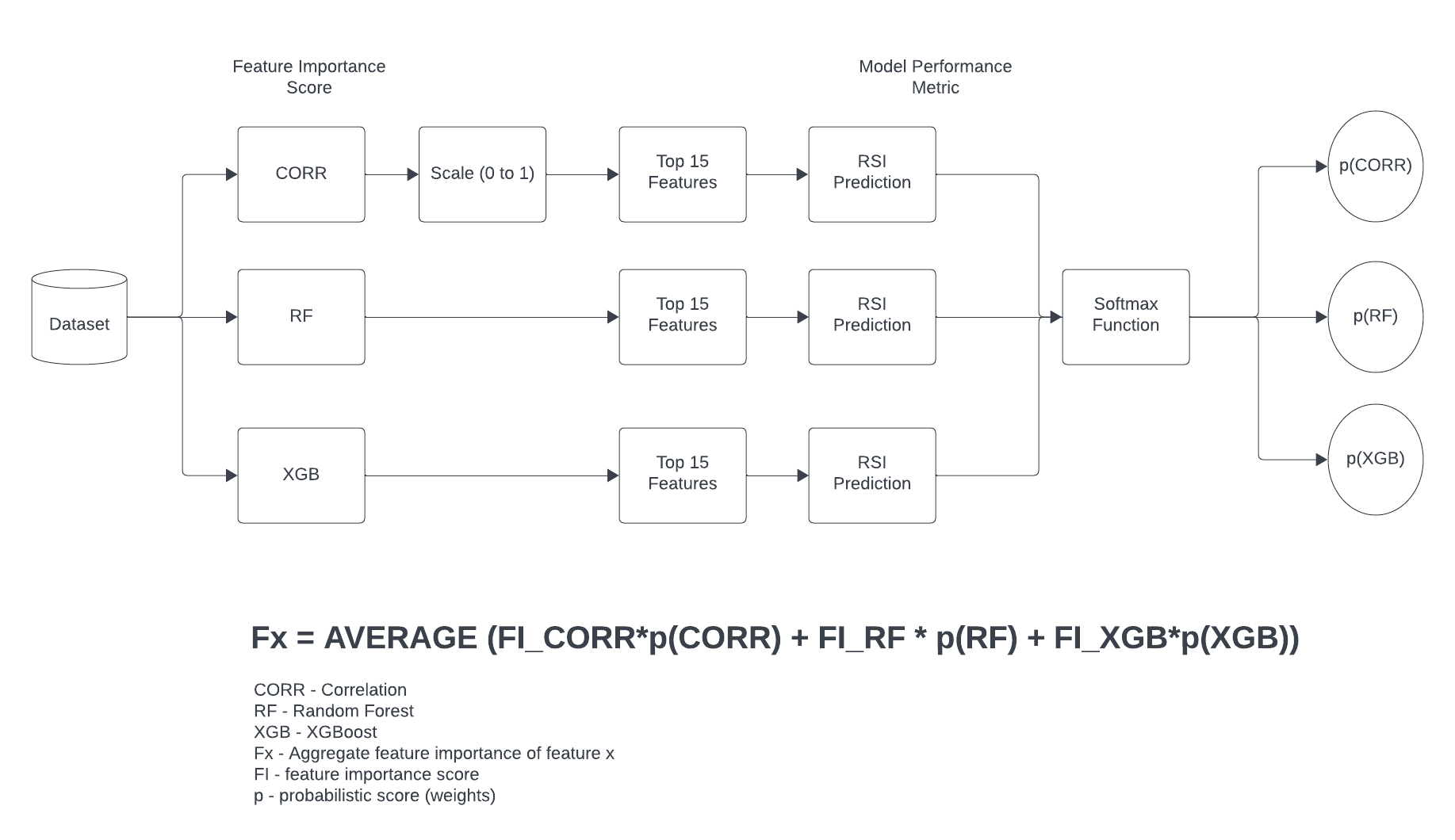


Fig.S1 Feature aggregation for CORR, RF, and XGB. Image was generated by LucidChart by the authors.

# Appendix VII: RSI Feature importance


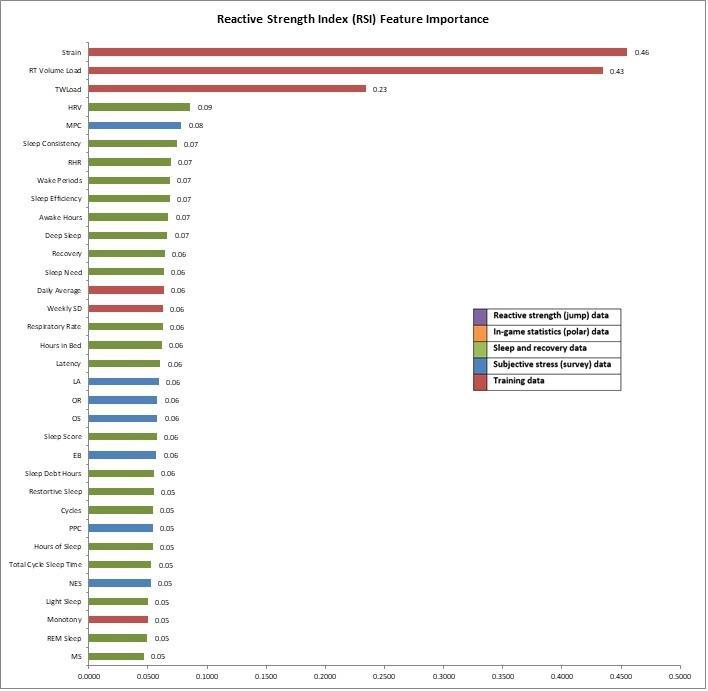


Fig. S2: Feature importance for the RSI.


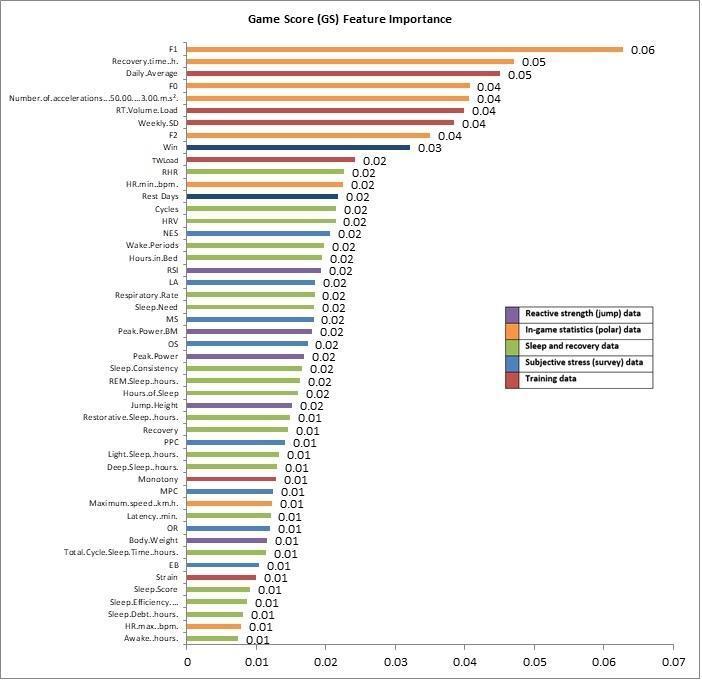


Fig. S3: Feature importance for the GS.


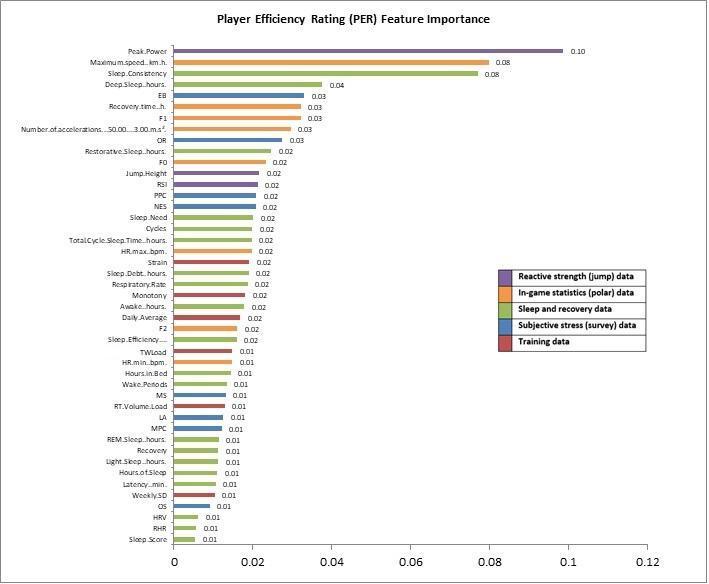


Fig. S4: Feature importance for the PER.

# Appendix VIII - Partial Dependency Plots


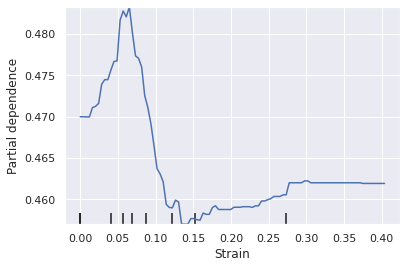

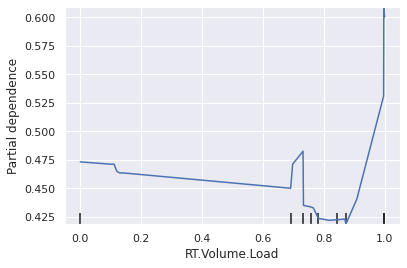

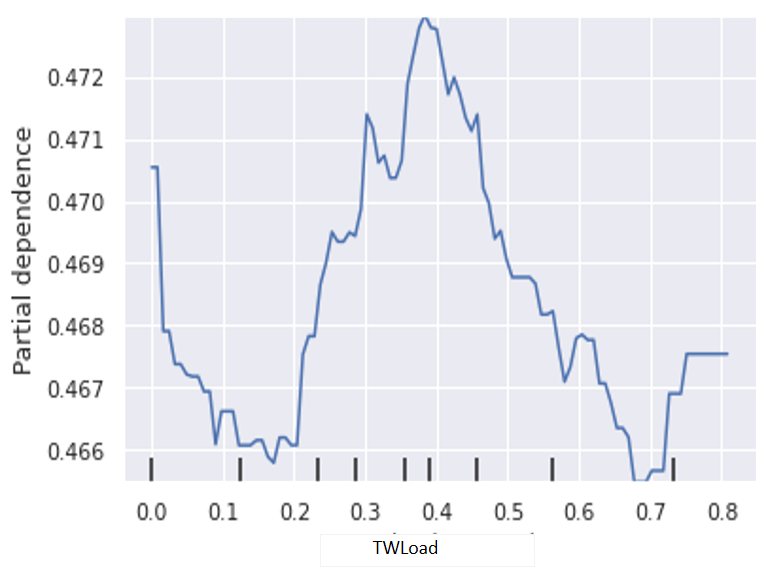


Fig. S5: PDPs for the three most significantly contributing features to RSI.


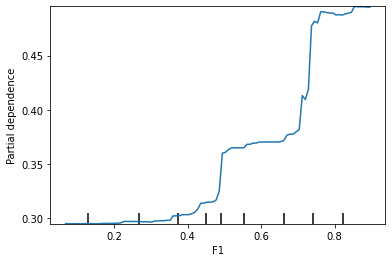

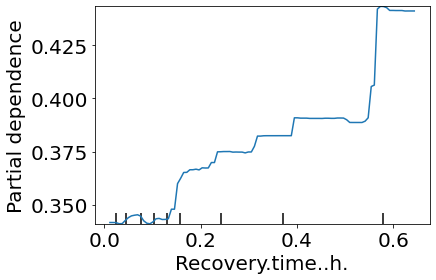

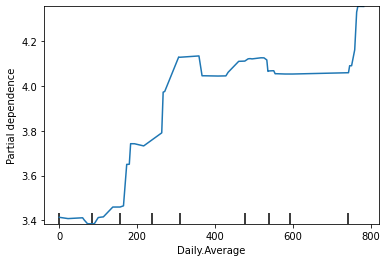


Fig. S6: PDPs for the three most significantly contributing features to the game score.


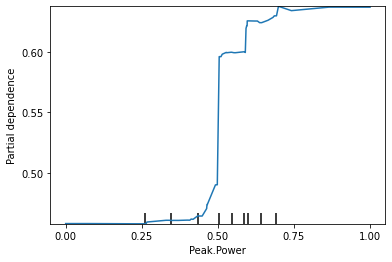

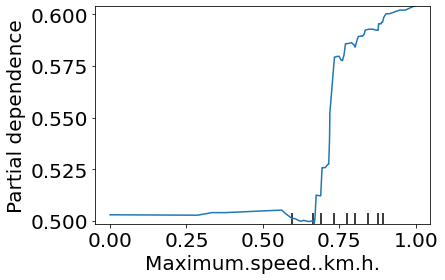

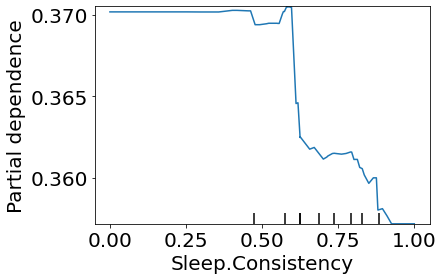


Fig. S7: PDPs for the three most significantly contributing features to PER.

# Appendix IX - Dashboard

We are currently using a Google Spreadsheet to give coaches feedback on athlete recovery and readiness (a snapshot is provided below, athlete name is covered). The future work will include providing weekly predictions to the coaches for them to make informed decisions.

**
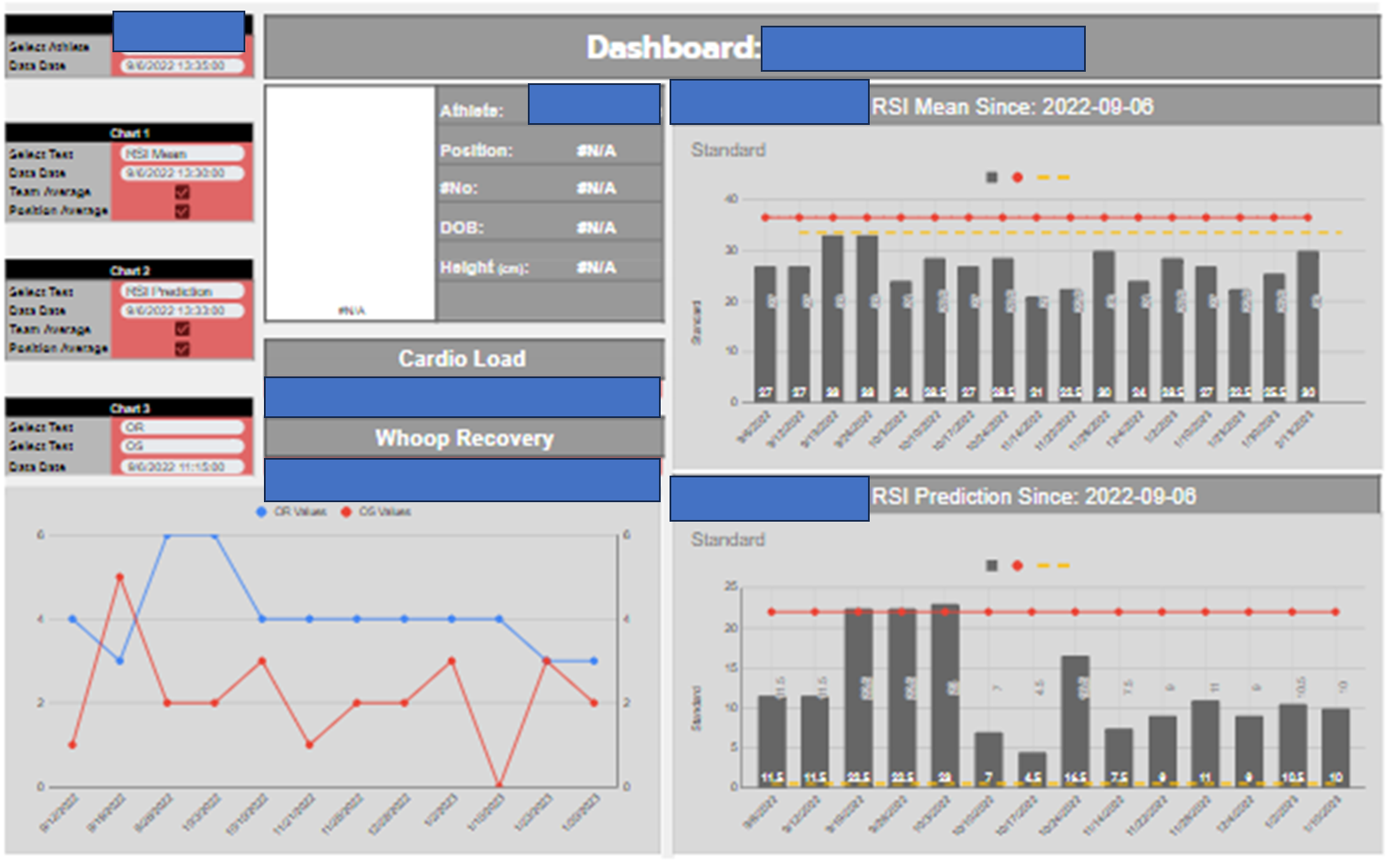
**

Fig. S8: Currently utilized Dashboard system by the data scientists and the team coaches.

# Appendix X - Time Series

We converted the dataset to make it suitable for predicting the RSI score for the following week (time-series prediction - one week ahead) using the XGBoost model. For each instance, for the independent features, we used the average value of the readings of week N, and the RSI score for week N+1 was used as the respective target feature value.

We also tried backtracking to assess how much data was sufficient to forecast the RSI score for the following weeks. We first tried predicting the RSI score for week 2 using data from week 1, then weeks 1 and 2 for predicting week 3, etc. The results are shown in Table S5. Data from weeks 11, 13, 15, 16, and 18 were not collected.

Table S5: Time series prediction for Week N + 1 based on past N week’s data.

|  | Accuracy | F1 score |
| --- | --- | --- |
| Week 2 (based on week 1) | 48.24% | 0.48 |
| Week 3 (based on week 1 and 2) | 42.45% | 0.41 |
| Week 4 (1-3) | 51.22% | 0.51 |
| Week 5 (1-4) | 51.67% | 0.52 |
| Week 6 (1-5) | 52.23% | 0.52 |
| Week 7 (1-6) | 55.71% | 0.56 |
| Week 8 (1-7) | 56.82% | 0.57 |
| Week 9 (1-8) | 54.28% | 0.54 |
| Week 10 (1-9) | 63.64% | 0.64 |
| Week 12 (1-11) | 68.26% | 0.68 |
| Week 14 (1-13) | 64.82% | 0.65 |
| Week 17 (1-16) | 70.91% | 0.71 |
| Week 19 (1-18) | 69.28% | 0.70 |

There was no pattern, consistency or precision observed in the predictions as some of the players contracted COVID during the season, leading to them missing some practice sessions and uneven patterns pre and post-COVID. We plan to incorporate the past few season’s data with the robust collection for the incoming year we developed to improve our methodology. This will be a future work where we will also include coaches' feedback in real-time.

# References:

(1) Nagarajan, R., & Li, L. Optimizing NBA player selection strategies based on salary and Statistics Analysis. *IEEE 15th Intl Conf on Dependable, Autonomic and Secure Computing, 15th Intl Conf on Pervasive Intelligence and Computing, 3rd Intl Conf on Big Data Intelligence and Computing and Cyber Science and Technology Congress(DASC/PiCom/DataCom/CyberSciTech)*, 2017. <https://doi.org/10.1109/dasc-picom-datacom-cyberscitec.2017.175> (last accessed 9/24/2023).

(2) Hamalian, Gregory. *Creating the Perfect NBA Team: A Look at PER and How It Affects Wins* [Unpublished Honors These]. Bridgewater State University, 2016. <https://vc.bridgew.edu/cgi/viewcontent.cgi?article=1184&context=honors_proj> (last accessed 9/24/2023)

(3) [2021-22 Women’s Basketball Cumulative Statistics. Available from:](https://www.zotero.org/google-docs/?S2K4XC)<https://sacredheartpioneers.com/sports/womens-basketball/stats> (Last accessed 9/24/2023).

(4) Yvette. *Beginner's guide on how to Calculate Player Efficiency Rating*. Watts Basketball, 2021. <https://wattsbasketball.com/blog/how-to-calculate-player-efficiency-rating> (last accessed 9/24/2023)

(5) Taneja, Shweta, Bhawna Suri, and Chirag Kothari. "Application of Balancing Techniques with Ensemble Approach for Credit Card Fraud Detection." International Conference on Computing, Power and Communication Technologies (GUCON). IEEE, 2019.

(6) More, Ajinkya. "Survey of resampling techniques for improving classification performance in unbalanced datasets." arXiv preprint arXiv:1608.06048 (2016).
